# Phase I Cancer Clinical Trials

**DOI:** 10.1038/sj.bjc.6603702

**Published:** 2007-04-17

**Authors:** J A Ledermann

**Affiliations:** 1 Cancer Research UK and University College London Cancer Trials Centre, London, UK

For many years, we have grown used to the infrequent arrival of new drugs to the market place. These represent the tip of the iceberg; many others undergo testing in phase I and II trials and fail to progress. The slow and steady stream of new drug development has been replaced by an enormous expansion of agents and therefore activity of phase I trials. Literally hundreds of new compounds have been generated over the last few years through expansion of knowledge of molecular biology and synthesis of agents to target recently identified cellular pathways and their receptors. The arrival of a book on Phase I Cancer Trials is timely.

The key strength and uniqueness of this book is, as its subtitle describes – a Practical Guide to Phase I Cancer Clinical Trials. The chapters take the reader in a clear and stepwise manner through the design, ethical aspects, writing of protocols and conduct of studies. The layout of the chapters is clear and very suitable for oncologists or scientists stepping into the world of first-in man studies. Of particular interest to this group are the chapters on how to write a protocol and file for regulatory submission. The latter deals with differences on the two sides of the Atlantic and the increasingly complex legal and regulatory processes involved in academic trials. The detailed and methodical approach is a clear testament to Elizabeth Eisenhauer and her colleagues’ considerable personal experience in dealing with this complex area.

I particularly liked the table inserted in the chapter on the preparation of the trial with lists of the Sponsor's responsibilities. A similar table on running a trial lists what to do if the unexpected happens. There is a substantial appendix with practical information – web resources and references and a sample protocol, consent form and other useful documentation. For completeness I would like to have seen included the NCI Common Terminology Criteria for Adverse Events v3.0.

Drug development and clinical trials are fundamental to the practice of medical oncology. Understanding the processes involved in developing and conducting clinical trials and active participation in them is an essential component of training and practice. Phase I clinical studies have hitherto been relatively straightforward, selecting an active drug based on the maximum tolerated dose and taking successful candidate agents into phase II trials. ‘More is better’ has been a paradigm that has only recently been challenged. This has created some difficulty for those working in early clinical trials. Maximum tolerated dose now is only one of a number of possible end points that include biological markers and surrogate measures of response. The clearly written chapter from Chris Twelves on the principles of pharmacokinetics and pharmacodynamics addresses this important area well, using up to date examples. Pharmacodynamics has become a more complex subject, now not only focusing on the effect of a drug on normal tissue, for example, bone marrow, but also its action on tumour tissue targets or other surrogate markers. Added to this are the growing number of studies that use positron emission tomography and magnetic resonance imaging. The latter, using dynamic contrast enhanced MRI, has been used extensively to evaluate vascular targeting agents. I would have welcomed even more detail on these topics, particularly tissue-based assays and surrogate end points.

No book on clinical trials is complete without a chapter on statistics, and Marc Buyse has dealt with this well. Classical design methods, their modification and newer approaches are explained clearly in a language that non-statisticians can easily understand.

This book is the first of its kind and should have a place on a library shelf. It is a very readable account of a complex area of oncology research. It is a handbook that I expect will soon be found lying on the desk of most trial units conducting early phase studies. It is likely to be referred to frequently, particularly as it is easy to dip into specific chapters to find the required information. It provides an excellent introduction and guide to the world of phase I cancer clinical trials.

